# Protective Effect of the HLA-DRB1*13:02 Allele in Japanese Rheumatoid Arthritis Patients

**DOI:** 10.1371/journal.pone.0099453

**Published:** 2014-06-09

**Authors:** Shomi Oka, Hiroshi Furukawa, Aya Kawasaki, Kota Shimada, Shoji Sugii, Atsushi Hashimoto, Akiko Komiya, Naoshi Fukui, Satoshi Ito, Tadashi Nakamura, Koichiro Saisho, Masao Katayama, Shinichiro Tsunoda, Hajime Sano, Kiyoshi Migita, Akiko Suda, Shouhei Nagaoka, Naoyuki Tsuchiya, Shigeto Tohma

**Affiliations:** 1 Clinical Research Center for Allergy and Rheumatology, Sagamihara Hospital, National Hospital Organization, Sagamihara, Japan; 2 Molecular and Genetic Epidemiology Laboratory, Faculty of Medicine, University of Tsukuba, Tsukuba, Japan; 3 Department of Rheumatology, Sagamihara Hospital, National Hospital Organization, Sagamihara, Japan; 4 Department of Rheumatology, Tokyo Metropolitan Tama Medical Center, Fuchu, Japan; 5 Department of Rheumatology, Niigata Rheumatic Center, Shibata, Japan; 6 Department of Rheumatology, Kumamoto Shinto General Hospital, Kumamoto, Japan; 7 Department of Orthopedics/Rheumatology, Miyakonojo Hospital, National Hospital Organization, Miyakonojo, Japan; 8 Department of Internal Medicine, Nagoya Medical Center, National Hospital Organization, Nagoya, Japan; 9 Division of Rheumatology, Department of Internal Medicine, Hyogo College of Medicine, Nishinomiya, Japan; 10 Clinical Research Center, Nagasaki Medical Center, National Hospital Organization, Omura, Japan; 11 Department of Rheumatology, Yokohama Minami Kyosai Hospital, Yokohama, Japan; 12 Center for Rheumatic Diseases, Yokohama City University Medical Center, Yokohama, Japan; Keio University School of Medicine, Japan

## Abstract

Rheumatoid arthritis (RA) is a chronic systemic inflammatory disease. Certain *HLA-DRB1* “shared-epitope” alleles are reported to be positively associated with increased RA susceptibility, whereas some of the other alleles may be negatively associated. However, studies on the latter are rare. Here, we focus on the protective effects of *DRB1* alleles in Japanese RA patients in an association study. Relative predispositional effects (RPE) were analyzed by sequential elimination of carriers of each allele with the strongest association. The protective effects of *DRB1* alleles were investigated in patients stratified according to whether they possessed anti-citrullinated peptide antibodies (ACPA). The DRB1*13:02 allele was found to be negatively associated with RA (*P* = 4.59×10^−10^, corrected *P* (*P*c) = 1.42×10^−8^, odds ratio [OR] 0.42, 95% CI 0.32–0.55, *P* [RPE] = 1.27×10^−6^); the genotypes DRB1*04:05/*13:02 and *09:01/*13:02 were also negatively associated with RA. The protective effect of *13:02 was also present in ACPA-positive patients (*P* = 3.95×10^−8^, *P*c = 1.22×10^−6^, OR 0.42, 95%CI 0.31–0.58) whereas *15:02 was negatively associated only with ACPA-negative RA (*P* = 8.87×10^−5^, *P*c = 0.0026, OR 0.26, 95%CI 0.12–0.56). Thus, this study identified a negative association of DRB1*13:02 with Japanese RA; our findings support the protective role of DRB1*13:02 in the pathogenesis of ACPA-positive RA.

## Introduction

Rheumatoid arthritis (RA) is a chronic systemic inflammatory disease that affects about 1% of the population. Its pathogenesis is multifactorial and disease susceptibility is associated with genetic and environmental factors [1], [2], [3]. Human Leukocyte Antigen (HLA) alleles are associated with RA in most ethnic groups and represent the strongest genetic risk factors for the disease. Most reports are of *HLA-DRB1* alleles positively associated with RA susceptibility. A conserved amino acid sequence at position 70–74 (QKRAA, RRRAA, or QRRAA) in the HLA-DRβ chain is shared between the RA susceptibility-associated *DRB1* alleles; this was designated the “shared epitope” (SE) [4]. A gene dosage effect was noted in the associations of *HLA-DRB1* alleles with susceptibility to RA in that homozygosity for susceptibility alleles does confer higher disease risk than heterozygosity for these alleles.

The presence of anti-citrullinated peptide antibodies (ACPA) is associated with RA with higher specificity than rheumatoid factor; thus, ACPA is thought to play some role in the pathogenesis of RA, especially as SE alleles are strongly associated with ACPA-positive RA but only relatively weakly with ACPA-negative RA [5]. Several studies have found that DRB1*04:01 and *04:05, both SE alleles, were mainly associated with RA in European and East Asian populations, respectively.

As well as associations with disease susceptibility, some *DRB1* alleles are reported to be negatively associated with RA. An amino acid sequence (DERAA) at position 70–74 [6], isoleucine at position 67 (I67) [7], aspartic acid at position 70 (D70) [8], or a conserved amino acid sequence at position 71–74 (S1; ARAA or ERAA) [9], [10] in the HLA-DRβ chain seem to be protective in European populations. It was also reported that DRB1*13 alleles are negatively associated with ACPA-positive and -negative RA in European populations [11]. A meta-analysis concluded that DRB1*13:01 was protective against ACPA-positive RA in European populations [12]. However, there are very few studies on the protective effects of *DRB1* alleles in Japanese patients, although reduced frequencies of some *DRB1* alleles have been reported in Asian RA [13], [14], [15], [16], [17]. In this study, we focus on the protective effects of *HLA-DRB1* alleles in Japanese RA patients with or without ACPA.

## Materials and Methods

### Patients and controls

RA patients (n = 1480) were recruited at Sagamihara Hospital, Tama Medical Center, Nagoya Medical Center, Nagasaki Medical Center, Yokohama Minami Kyosai Hospital, Kumamoto Center for Arthritis and Rheumatology, Miyakonojo Hospital, Niigata Rheumatic Center, and Hyogo College of Medicine. Of these 1480 RA patients, 919 were ACPA-positive and 110 were ACPA-negative. ACPA data were not available for the remaining 451 patients. Healthy controls (n = 800; mean age ± SD, 36.7±10.7 years, 238 male [30.1%]) were recruited at Sagamihara Hospital and University of Tokyo, or by the Pharma SNP Consortium (Tokyo, Japan) [18]. All patients and healthy individuals were native Japanese living in Japan. All patients with RA fulfilled the 1987 American College of Rheumatology criteria for RA [19]. Rheumatoid factor and ACPA were detected using the N-latex RF kit (Siemens Healthcare Diagnostics, München, Germany) and the Mesacup-2 test CCP (Medical & Biological Laboratories, Nagoya, Japan), respectively. This study was reviewed and approved by the Research Ethics Committees of each participating institute: Nagasaki Medical Center Research Ethics Committee, Yokohama Minami Kyosai Hospital Research Ethics Committee, Tama Medical Center Research Ethics Committee, University of Tsukuba Research Ethics Committee, Miyakonojo Hospital Research Ethics Committee, Kumamoto Center for Arthritis and Rheumatology Research Ethics Committee, Niigata Rheumatic Center Research Ethics Committee, Hyogo College of Medicine Research Ethics Committee, and the University of Tokyo Research Ethics Committee. Written informed consent was obtained from all study participants. This study was conducted in accordance with the principles expressed in the Declaration of Helsinki.

### Genotyping

Genotyping of *HLA-DRB1* was performed by a polymerase chain reaction technique using sequence-specific oligonucleotide probes (WAKFlow HLA typing kits, Wakunaga, Hiroshima, Japan), using a Bio-Plex 200 system (Bio-Rad, Hercules, CA), or using MPH-2 High Resolution HLA typing kits (Wakunaga) for four-digit allele typing. The following *DRB1* alleles contain the SE [4]: *01:01, *04:01, *04:04, *04:05, *04:10, *10:01, *14:02, and *14:06. *DRB1* allele groups, D70, I67, S1, and DERAA, were reported to be protective in European populations [6], [7], [8], [9], [10]; the protective effects of these allele groups in Japanese were validated in this study. *DRB1* alleles containing D70 [8] are *07:01, *08:02, *08:03, *08:09, *08:23, *11:01, *11:06, *12:01, *12:02, *12:05, *13:01, *13:02, *13:07, *14:03, *14:12, and *16:02. DRB1 alleles containing I67 [7] are *07:01, *08:03, *08:23, *12:01, *12:05, *13:01, *13:02, *14:45, *15:01, *15:02, and *15:11. DRB1 alleles containing DERAA [6] are the same as DRB1*13 (i.e. *13:01, and *13:02). Finally, *DRB1* alleles containing S1 [20] are *13:01, *13:02, *15:01, and *15:02. Results of *DRB1* genotyping for some of the healthy controls were reported previously [14]. Some of the RA patients were also included in another study which reported on susceptibility effects for interstitial lung disease or positivity for autoantibodies [21], [22], [23]. *HLA-DRB1* genotype of each subject was not deposited in publicly available resources.

### Statistical analysis

The exact tests for deviation from Hardy-Weinberg equilibrium were conducted by the Markov chain method under the condition of 10000 each of dememorization, batches, and iterations per batch (Genepop on the web; http://genepop.curtin.edu.au/) [24]. Differences of allele carrier frequencies, genotype frequencies or amino acid residue carrier frequencies were analyzed by Fisher's exact test using 2×2 contingency tables. In order to estimate the protective effects of alleles in multi-allelic locus on individuals for RA, differences of allele carrier frequencies, or amino acid residue carrier frequencies were analyzed under the dominant model. Adjustment for multiple comparisons was performed using the Bonferroni method. *P*c values were calculated by multiplying the *P* value by the number of alleles or amino acid residues tested.

Alleles with low carrier frequencies in RA patients may not be detectably protective because predisposing SE alleles with higher carrier frequencies could obscure their influence. To investigate the protective effects of HLA alleles, relative predispositional effects (RPE) were analyzed by sequential elimination of carriers of each allele with the strongest association [25]. In order to obtain an accurate estimate of the effects of alleles other than SE, analyses of these alleles in RA patients were also stratified in the following manner: For SE-negative subjects, the effect in “*A*/*A*” and “*A*/other than SE or *A*” genotype groups was investigated using “other than SE or *A*/other than SE or *A*” genotype group as the reference. For SE-positive subjects, the effect of “SE/*A*” genotype group was analyzed using “SE/other than *A*” genotype group as the reference. The protective effects of the *13:02 allele were confirmed in the presence of predisposing allele “*B*”. The effect in “*B*/*13:02” genotype group was investigated using the “*B*/other than *13:02” genotype group as the reference. The protective effects of the *15:02 allele were confirmed in the analysis of “*B*/*15:02” using the “*B*/other than *15:02” genotype group as the reference in the same manner.

## Results

### Characteristics of RA patients

Characteristics of ACPA-positive [ACPA(+)] and ACPA-negative [ACPA(−)] RA patients are given in Table 1. The proportion of rheumatoid factor-positive patients in the ACPA(+) group was higher than in ACPA(−) RA. There were no significant differences in terms of mean age, percentage of males, age at onset, or Steinbrocker stage [26] between ACPA(+) and ACPA(-) patients.

**Table 1:** Characteristics of the RA patients studied.

	RA	ACPA(+) RA	ACPA(−) RA	*P*
Number	1480	919	110	
Mean age, years (SD)	63.9 (12.2)	63.7 (12.2)	63.4 (12.3)	0.8582*
Male, n (%)	272 (19.0)	171 (18.7)	21 (19.3)	0.8969
Age at onset, years (SD)	49.3 (14.4)	49.2 (14.2)	50.1 (16.7)	0.6092*
Steinbrocker stage III and IV, n (%)	560 (37.8)	521 (56.7)	45 (40.9)	0.0703
Rheumatoid factor positive, n (%)	1002 (67.7)	826 (89.9)	40 (36.4)	9.39×10^−37^

RA: rheumatoid arthritis, ACPA: anti-citrullinated peptide antibody, ACPA(+): ACPA-positive, ACPA(−): ACPA-negative. Association was tested by Fisher's exact test using 2×2 contingency tables or Student's t-test. *Student's t-test was employed.

### Reduced HLA-DRB1*13:02 allele carrier frequency in Japanese RA

*HLA-DRB1* genotyping was performed in 1480 RA patients and 800 healthy controls to compare HLA allele carrier frequencies (Table 2). No deviation from Hardy-Weinberg equilibrium was observed in the controls (*P* = 0.6329), though a deviation was detected in the RA patients (*P*<0.0001). A strong positive association between the frequency of DRB1*04 and RA (*P* = 1.00×10^−22^, Corrected *P* [*P*c] = 1.31×10^−21^, odds ratio [OR] 2.40, 95% confidence interval [CI] 2.01–2.86, Table 2) was confirmed. Additionally, DRB1*13 (i.e. the DERAA allele group) was found to be negatively associated with RA (*P* = 4.69×10^−11^, *P*c = 6.10×10^−10^, OR 0.41, 95% CI 0.31–0.53). The D70, I67, and S1 allele groups were also negatively associated with RA (D70: *P* = 1.15×10^−20^, OR 0.43, 95% CI 0.36–0.52; I67: *P* = 3.67×10^−13^, OR 0.52, 95% CI 0.44–0.62; S1: *P* = 2.51×10^−9^, OR 0.59, 95% CI 0.49–0.70). Finally, a predisposing association was confirmed between SE and RA (*P* = 2.35×10^−45^, OR 3.58, 95% CI 2.99–4.29).

**Table 2:** *HLA-DRB1* allele carrier frequency in RA patients and controls.

	RA (n = 1480)	Control (n = 800)	*P*	OR	*P*c	95%CI	*P* (RPE)
DRB1*04	901 (60.9)	315 (39.4)	1.00×10^−22^	2.40		(2.01–2.86)	
DRB1*08	188 (12.7)	181 (22.6)	2.02×10^−9^	0.50		(0.40–0.62)	
DRB1*12	143 (9.7)	87 (10.9)	0.3820	0.88		(0.66–1.16)	
DRB1*13	112 (7.6)	134 (16.8)	4.69×10^−11^	0.41		(0.31–0.53)	
DRB1*14	180 (12.2)	143 (17.9)	0.0003	0.64		(0.50–0.81)	
DRB1*15	405 (27.4)	262 (32.8)	0.0080	0.77		(0.64–0.93)	
SE	1035 (69.9)	315 (39.4)	2.35×10^−45^	3.58		(2.99–4.29)	
D70	503 (34.0)	434 (54.3)	1.15×10^−20^	0.43		(0.36–0.52)	
I67	691 (46.7)	501 (62.6)	3.67×10^−13^	0.52		(0.44–0.62)	
S1	504 (34.1)	375 (46.9)	2.51×10^−9^	0.59		(0.49–0.70)	
DRB1*01:01	210 (14.2)	83 (10.4)	0.0104	1.43	0.3239	(1.09–1.87)	3.75×10^−5^
DRB1*03:01	2 (0.1)	0 (0.0)	0.5443	2.71	NS	(0.13–56.46)	
DRB1*04:01	84 (5.7)	17 (2.1)	4.30×10^−5^	2.77	0.0013	(1.63–4.70)	0.0002
DRB1*04:03	38 (2.6)	42 (5.3)	0.0012	0.48	0.0374	(0.30–0.74)	
DRB1*04:04	5 (0.3)	4 (0.5)	0.7280	0.67	NS	(0.18–2.52)	
DRB1*04:05	738 (49.9)	185 (23.1)	1.41×10^−36^	3.31	4.37×10^−35^	(2.73–4.01)	1.41×10^−36^
DRB1*04:06	58 (3.9)	59 (7.4)	0.0005	0.51	0.0148	(0.35–0.74)	
DRB1*04:07	4 (0.3)	15 (1.9)	0.0001	0.14	0.0035	(0.05–0.43)	
DRB1*04:10	70 (4.7)	21 (2.6)	0.0136	1.84	0.4224	(1.12–3.02)	0.0109
DRB1*07:01	10 (0.7)	7 (0.9)	0.6155	0.77	NS	(0.29–2.03)	
DRB1*08:02	56 (3.8)	61 (7.6)	0.0001	0.48	0.0042	(0.33–0.69)	
DRB1*08:03	135 (9.1)	124 (15.5)	8.60×10^−6^	0.55	0.0003	(0.42–0.71)	
DRB1*08:09	1 (0.1)	2 (0.3)	0.2829	0.27	NS	(0.02–2.98)	
DRB1*08:23	1 (0.1)	0 (0.0)	1.0000	1.62	NS	(0.07–39.89)	
DRB1*09:01	423 (28.6)	213 (26.6)	0.3282	1.10	NS	(0.91–1.34)	7.32×10^−5^
DRB1*10:01	25 (1.7)	2 (0.3)	0.0017	6.86	0.0536	(1.62–29.02)	0.0128
DRB1*11:01	40 (2.7)	33 (4.1)	0.0803	0.65	NS	(0.40–1.03)	0.0236
DRB1*12:01	95 (6.4)	58 (7.3)	0.4830	0.88	NS	(0.63–1.23)	
DRB1*12:02	50 (3.4)	29 (3.6)	0.8105	0.93	NS	(0.58–1.48)	
DRB1*13:01	5 (0.3)	8 (1.0)	0.0752	0.34	NS	(0.11–1.03)	
DRB1*13:02	107 (7.2)	126 (15.8)	4.59×10^−10^	0.42	1.42×10^−8^	(0.32–0.55)	1.27×10^−6^
DRB1*14:02	2 (0.1)	0 (0.0)	0.5443	2.71	NS	(0.13–56.46)	
DRB1*14:03	32 (2.2)	38 (4.8)	0.0009	0.44	0.0271	(0.27–0.72)	
DRB1*14:04	0 (0.0)	3 (0.4)	0.0431	0.08	NS	(0.00–1.49)	
DRB1*14:05	28 (1.9)	35 (4.4)	0.0011	0.42	0.0348	(0.25–0.70)	
DRB1*14:06	45 (3.0)	22 (2.8)	0.7952	1.11	NS	(0.66–1.86)	0.0041
DRB1*14:07	2 (0.1)	2 (0.3)	0.6159	0.54	NS	(0.08–3.84)	
DRB1*14:54	76 (5.1)	45 (5.6)	0.6254	0.91	NS	(0.62–1.33)	
DRB1*15:01	178 (12.0)	107 (13.4)	0.3537	0.89	NS	(0.68–1.14)	
DRB1*15:02	233 (15.7)	168 (21.0)	0.0019	0.70	0.0574	(0.56–0.88)	
DRB1*16:02	17 (1.1)	15 (1.9)	0.1912	0.61	NS	(0.30–1.22)	

RA: rheumatoid arthritis, OR: odds ratio, CI: confidence interval, *P*c: corrected *P* value, NS: not significant, RPE: relative predispositional effects. Allele carrier frequencies are shown in parenthesis (%). Association was tested by Fisher's exact test using 2×2 contingency tables. RPE were tested by sequential elimination of carriers of each of the alleles DRB1*04:05, *13:02, *04:01, *09:01, *01:01, *14:06, *10:01, *04:10, and *11:01. Allele groups SE, D70, I67, and S1 were as defined in the Materials and Methods section. *DRB1* alleles encoding the DERAA were the same as DRB1*13 (i.e. *13:01 and *13:02).

We further explored associations between these *DRB1* alleles and RA by high-resolution typing, using RPE testing [25] (Table 2). RPE were analyzed by sequential elimination of carriers of each allele with the strongest association (Table 2, right column). The prime strongest association was between DRB1*04:05 and RA (*P* = 1.41×10^−36^, *P*c = 4.37×10^−35^, OR 3.31, 95% CI 2.73–4.01). Thus, a second round of comparisons was conducted after the elimination of DRB1*04:05 carriers, revealing the next strongest association to be between DRB1*13:02 and RA (*P* = 1.27×10^−6^, *P*c = 3.68×10^−5^). A third round after the elimination of both DRB1*04:05 or *13:02 carriers now showed the strongest association of RA with DRB1*04:01 (*P* = 0.0002, *P*c = 0.0065). Further rounds after elimination of DRB1*04:05, *13:02 and *04:01 carriers revealed associations between the remaining *DRB1* alleles and RA, particularly for DRB1*09:01 (*P* = 7.32×10^−5^, *P*c = 0.0020), *01:01 (*P* = 3.75×10^−5^, *P*c = 0.0010), *14:06 (*P* = 0.0041, *P*c = 0.0995), *10:01 (*P* = 0.0128, *P*c = 0.2936), *04:10 (*P* = 0.0109, *P*c = 0.2399), and *11:01 (*P* = 0.0236, *P*c = 0.4948). The results from association studies under the recessive and the allele models were represented in Table S1 and S2, respectively. Similar tendencies were observed in these analyses. We therefore focused on the *DRB1* allele with the most significantly reduced allele carrier frequency, namely DRB1*13:02.

### Protective effects of the *13:02 allele against RA in both SE-positive and -negative subjects

In order to obtain an accurate estimate of the effects of alleles other than SE, associations were estimated in subjects stratified into those with or without SE (Table 3). Although DRB1*09 (*P* = 1.44×10^−8^, OR 2.15, 95% CI 1.65–2.81) predisposes to RA in SE-negative people, *04 other than SE (*04:03, *04:06, *04:07: SE negative, *P* = 0.0055, OR 0.58, 95% CI 0.39–0.85; SE positive, *P* = 0.0010, OR 0.45, 95% CI 0.29–0.71), *13 (*13:01, *13:02: SE negative, *P* = 2.43×10^−5^, OR 0.45, 95% CI 0.31–0.66; SE positive, *P* = 0.0235, OR 0.58, 95% CI 0.37–0.91), and D70 (SE negative, *P* = 0.0004, OR 0.61, 95% CI 0.47–0.80; SE positive, *P* = 0.0002, OR 0.59, 95% CI 0.45–0.78) were negatively associated with RA in both SE-positive and -negative individuals. DRB1*08 (*P* = 0.0018, OR 0.51, 95% CI 0.34–0.77) alleles were negatively associated with RA in SE-positive people. I67 (*P* = 0.0027, OR 0.64, 95% CI 0.48–0.86) and S1 (*P* = 0.0012, OR 0.65, 95% CI 0.50–0.84) alleles were negatively associated with RA in SE-negative subjects. However, D70 alleles other than *13:02 were negatively associated with RA in SE-positive (*P* = 0.0092, OR 0.67, 95% CI 0.50–0.90) but not in SE-negative individuals. I67 alleles other than *13:02 and S1 alleles other than *13:02 did not have any negative associations. These data suggest that the negative associations of D70, I67 and S1 alleles with RA in SE-negative subjects were mainly mediated by *13:02, although the negative association of D70 in SE-positive people was due to *08 alleles. Thus, *13:02 was negatively associated with RA in SE-negative people and relatively weakly also in SE-positive subjects.

**Table 3:** *HLA-DRB1* allele carrier frequency in RA patients and controls in subjects stratified for the presence of SE.

		RA (n = 1480)	Control (n = 800)	*P*	OR	95%CI
*03	SE negative	1 (0.2)	0 (0.0)	0.4785	3.28	(0.13–80.65)
	SE positive	1 (0.1)	0 (0.0)	1.0000	0.91	(0.04–22.52)
*04 other than SE	SE negative	46 (10.3)	81 (16.7)	0.0055	0.58	(0.39–0.85)
	SE positive	54 (5.2)	34 (10.8)	0.0010	0.45	(0.29–0.71)
*07	SE negative	5 (1.1)	5 (1.0)	1.0000	1.09	(0.31–3.79)
	SE positive	5 (0.5)	2 (0.6)	0.6680	0.76	(0.15–3.93)
*08	SE negative	114 (25.6)	140 (28.9)	0.2703	0.85	(0.64–1.13)
	SE positive	74 (7.1)	41 (13.0)	0.0018	0.51	(0.34–0.77)
*09	SE negative	230 (51.7)	161 (33.2)	1.44×10^−8^	2.15	(1.65–2.81)
	SE positive	193 (18.6)	52 (16.5)	0.4051	1.16	(0.83–1.62)
*11	SE negative	17 (3.8)	29 (6.0)	0.1336	0.62	(0.34–1.15)
	SE positive	23 (2.2)	4 (1.3)	0.3635	1.77	(0.61–5.15)
*12	SE negative	63 (14.2)	66 (13.6)	0.8496	1.05	(0.72–1.52)
	SE positive	80 (7.7)	21 (6.7)	0.6248	1.17	(0.71–1.93)
*13	SE negative	48 (10.8)	102 (21.0)	2.43×10^−5^	0.45	(0.31–0.66)
	SE positive	64 (6.2)	32 (10.2)	0.0235	0.58	(0.37–0.91)
*14 other than SE	SE negative	63 (14.2)	89 (18.4)	0.0918	0.73	(0.52–1.04)
	SE positive	72 (7.0)	33 (10.5)	0.0537	0.64	(0.41–0.98)
*15	SE negative	182 (40.9)	207 (42.7)	0.5950	0.93	(0.72–1.21)
	SE positive	223 (21.5)	55 (17.5)	0.1305	1.30	(0.94–1.80)
*16	SE negative	10 (2.2)	9 (1.9)	0.8174	1.22	(0.49–3.02)
	SE positive	7 (0.7)	6 (1.9)	0.0900	0.35	(0.12–1.05)
*13:01	SE negative	1 (0.2)	7 (1.4)	0.0712	0.15	(0.02–1.26)
	SE positive	4 (0.4)	1 (0.3)	1.0000	1.22	(0.14–10.94)
*13:02	SE negative	47 (10.6)	95 (19.6)	0.0001	0.48	(0.33–0.71)
	SE positive	60 (5.8)	31 (9.8)	0.0148	0.56	(0.36–0.89)
D70	SE negative	242 (54.4)	320 (66.0)	0.0004	0.61	(0.47–0.80)
	SE positive	261 (25.2)	114 (36.2)	0.0002	0.59	(0.45–0.78)
I67	SE negative	301 (67.6)	371 (76.5)	0.0027	0.64	(0.48–0.86)
	SE positive	390 (37.7)	130 (41.3)	0.2614	0.86	(0.67–1.11)
S1	SE negative	217 (48.8)	288 (59.4)	0.0012	0.65	(0.50–0.84)
	SE positive	287 (27.7)	87 (27.6)	1.0000	1.01	(0.76–1.33)
D70 other than *13:02	SE negative	212 (47.6)	253 (52.2)	0.1892	0.83	(0.64–1.08)
	SE positive	201 (19.4)	83 (26.3)	0.0092	0.67	(0.50–0.90)
I67 other than *13:02	SE negative	280 (62.9)	310 (63.9)	0.7852	0.96	(0.73–1.25)
	SE positive	330 (31.9)	99 (31.4)	0.8904	1.02	(0.78–1.34)
S1 other than *13:02	SE negative	182 (40.9)	213 (43.9)	0.3536	0.88	(0.68–1.15)
	SE positive	227 (21.9)	56 (17.8)	0.1147	1.30	(0.94–1.80)

RA: rheumatoid arthritis, SE: Shared epitope, OR: odds ratio, CI: confidence interval, Allele carrier frequencies are shown in parenthesis (%). Association was tested by Fisher's exact test using 2×2 contingency tables. SE negative: “*A*/*A*” or “*A*/other than SE or *A*” vs. “other than SE or *A*/other than SE or *A*”. SE positive: “SE/*A*” vs. “SE/other than *A*”. Allele groups SE, D70, I67, and S1 were as defined in the Materials and Methods section.

The protective effects of the *13:02 allele were analyzed in the presence of predisposing alleles (Table 4). Although *04:05 and *09:01 are positively associated with RA in Japanese, the risk of disease in people carrying these alleles was decreased in heterozygotes also carrying *13:02 (*04:05: *P* = 0.0168, OR = 0.51, 95% CI 0.29–0.87; *09:01: *P* = 0.0004, OR = 0.27, 95% CI 0.13–0.55).

**Table 4:** *HLA-DRB1* genotype frequency in RA patients and controls.

	RA (n = 1480)	Control (n = 800)	*P*	OR	95%CI
*04:05/*13:02	45 (6.1)	21 (11.4)	0.0168	0.51	(0.29–0.87)
*04:01/*13:02	6 (7.1)	2 (11.8)	0.6190	0.58	(0.11–3.14)
*09:01/*13:02	12 (2.8)	21 (9.9)	0.0004	0.27	(0.13–0.55)
*01:01/*13:02	5 (2.4)	3 (3.6)	0.6917	0.65	(0.15–2.79)

RA: rheumatoid arthritis, OR: odds ratio, CI: confidence interval, Allele carrier frequencies are shown in parenthesis (%). Association was tested by Fisher's exact test using 2×2 contingency tables. Comparison: “*B*/*13:02” vs. *B*/other than *13:02”.

Although the age at RA onset in *04:05 allele carriers was lower than in non-carriers (mean age ± standard deviation [SD] [years], carriers vs. non-carriers, 48.2±13.8 vs. 50.5±14.9, *P* = 0.0070) the age at onset in people with *13:02 or *01:01 was higher than in non-carriers (53.8±14.0 vs. 48.9±14.4, *P* = 0.0027, and 52.9±13.3 vs. 48.7±14.5, *P* = 0.0021, respectively) (Table S3).

### Protective effects of *13:02 against ACPA(+) RA and *15:02 against ACPA(−) RA

Predisposing effects of the *04:05 allele were confirmed in ACPA(+) RA (Table 5, *P* = 3.64×10^−35^, *P*c = 1.13×10^−33^, OR 3.59, 95% CI 2.91–4.42), whereas DRB1*13:02 was negatively associated with ACPA(+) RA (*P* = 3.95×10^−8^, *P*c = 1.22×10^−6^, OR 0.42, 95% CI 0.31–0.58). The DERAA allele group was still negatively associated with RA even when only ACPA(+) patients were considered (*P* = 2.05×10^−9^, OR 0.40, 95% CI 0.29–0.54). D70, I67, and S1 were also negatively associated with ACPA(+) RA (D70: *P* = 5.78×10^−21^, OR 0.39, 95% CI 0.32–0.48; I67: *P* = 3.66×10^−12^, OR 0.50, 95% CI 0.42–0.61; S1: *P* = 2.31×10^−8^, OR 0.57, 95% CI 0.47–0.70), and the predisposing association was confirmed between SE and ACPA(+) RA (*P* = 1.16×10^−48^, OR 4.41, 95% CI 3.59–5.41).

**Table 5:** *HLA-DRB1* allele carrier frequency in ACPA(+) and ACPA(−) RA patients and controls.

	ACPA(+) RA	ACPA(−) RA	Control	ACPA(+) RA				ACPA(−) RA			
	(n = 919)	(n = 110)	(n = 800)	*P*	OR	*P*c	95%CI	*P*	OR	*P*c	95%CI
DRB1*01:01	146 (15.9)	10 (9.1)	83 (10.4)	0.0008	1.63	0.0251	(1.22–2.18)	0.8664	0.86	NS	(0.43–1.72)
DRB1*03:01	1 (0.1)	1 (0.9)	0 (0.0)	1.0000	2.61	NS	(0.11–64.28)	0.1209	21.93	NS	(0.89–541.76)
DRB1*04:01	65 (7.1)	4 (3.6)	17 (2.1)	1.12×10^−6^	3.51	3.48×10^−5^	(2.04–6.03)	0.3069	1.74	NS	(0.57–5.26)
DRB1*04:03	22 (2.4)	1 (0.9)	42 (5.3)	0.0020	0.44	0.0632	(0.26–0.75)	0.0512	0.17	NS	(0.02–1.22)
DRB1*04:04	4 (0.4)	0 (0.0)	4 (0.5)	1.0000	0.87	NS	(0.22–3.49)	1.0000	0.80	NS	(0.04–14.98)
DRB1*04:05	477 (51.9)	38 (34.5)	185 (23.1)	3.64×10^−35^	3.59	1.13×10^−33^	(2.91–4.42)	0.0126	1.75	0.3667	(1.15–2.69)
DRB1*04:06	32 (3.5)	9 (8.2)	59 (7.4)	0.0003	0.45	0.0106	(0.29–0.70)	0.7014	1.12	NS	(0.54–2.33)
DRB1*04:07	3 (0.3)	0 (0.0)	15 (1.9)	0.0017	0.17	0.0514	(0.05–0.59)	0.2387	0.23	NS	(0.01–3.86)
DRB1*04:10	46 (5.0)	3 (2.7)	21 (2.6)	0.0122	1.95	0.3768	(1.16–3.30)	1.0000	1.04	NS	(0.31–3.55)
DRB1*07:01	7 (0.8)	0 (0.0)	7 (0.9)	0.7956	0.87	NS	(0.30–2.49)	1.0000	0.48	NS	(0.03–8.44)
DRB1*08:02	27 (2.9)	10 (9.1)	61 (7.6)	1.39×10^−5^	0.37	0.0004	(0.23–0.58)	0.5703	1.21	NS	(0.60–2.44)
DRB1*08:03	80 (8.7)	13 (11.8)	124 (15.5)	1.83×10^−5^	0.52	0.0006	(0.39–0.70)	0.3931	0.73	NS	(0.40–1.34)
DRB1*08:09	0 (0.0)	0 (0.0)	2 (0.3)	0.2164	0.17	NS	(0.01–3.62)	1.0000	1.45	NS	(0.07–30.30)
DRB1*08:23	1 (0.1)	0 (0.0)	0 (0.0)	1.0000	2.61	NS	(0.11–64.28)				
DRB1*09:01	252 (27.4)	30 (27.3)	213 (26.6)	0.7440	1.04	NS	(0.84–1.29)	0.9086	1.03	NS	(0.66–1.62)
DRB1*10:01	15 (1.6)	0 (0.0)	2 (0.3)	0.0054	6.62	0.1669	(1.51–29.04)	1.0000	1.45	NS	(0.07–30.30)
DRB1*11:01	26 (2.8)	4 (3.6)	33 (4.1)	0.1463	0.68	NS	(0.40–1.14)	1.0000	0.88	NS	(0.30–2.52)
DRB1*12:01	53 (5.8)	14 (12.7)	58 (7.3)	0.2379	0.78	NS	(0.53–1.15)	0.0579	1.87	NS	(1.00–3.47)
DRB1*12:02	27 (2.9)	5 (4.5)	29 (3.6)	0.4963	0.80	NS	(0.47–1.37)	0.5922	1.27	NS	(0.48–3.34)
DRB1*13:01	1 (0.1)	1 (0.9)	8 (1.0)	0.0149	0.11	0.4632	(0.01–0.86)	1.0000	0.91	NS	(0.11–7.33)
DRB1*13:02	67 (7.3)	14 (12.7)	126 (15.8)	3.95×10^−8^	0.42	1.22×10^−6^	(0.31–0.58)	0.4819	0.78	NS	(0.43–1.41)
DRB1*14:02	1 (0.1)	0 (0.0)	0 (0.0)	1.0000	2.61	NS	(0.11–64.28)				
DRB1*14:03	17 (1.8)	4 (3.6)	38 (4.8)	0.0008	0.38	0.0257	(0.21–0.67)	0.8088	0.76	NS	(0.26–2.16)
DRB1*14:04	0 (0.0)	0 (0.0)	3 (0.4)	0.1006	0.12	NS	(0.01–2.40)	1.0000	1.03	NS	(0.05–20.09)
DRB1*14:05	18 (2.0)	6 (5.5)	35 (4.4)	0.0048	0.44	0.1482	(0.25–0.78)	0.6219	1.26	NS	(0.52–3.07)
DRB1*14:06	32 (3.5)	3 (2.7)	22 (2.8)	0.4086	1.28	NS	(0.74–2.21)	1.0000	0.99	NS	(0.29–3.37)
DRB1*14:07	1 (0.1)	0 (0.0)	2 (0.3)	0.6007	0.43	NS	(0.04–4.80)	1.0000	1.45	NS	(0.07–30.30)
DRB1*14:54	43 (4.7)	13 (11.8)	45 (5.6)	0.3824	0.82	NS	(0.54–1.26)	0.0202	2.25	0.5861	(1.17–4.32)
DRB1*15:01	106 (11.5)	15 (13.6)	107 (13.4)	0.2711	0.84	NS	(0.63–1.13)	0.8824	1.02	NS	(0.57–1.83)
DRB1*15:02	145 (15.8)	7 (6.4)	168 (21.0)	0.0058	0.70	0.1797	(0.55–0.90)	8.87×10^−5^	0.26	0.0026	(0.12–0.56)
DRB1*16:02	10 (1.1)	2 (1.8)	15 (1.9)	0.2255	0.58	NS	(0.26–1.29)	1.0000	0.97	NS	(0.22–4.30)
SE	681 (74.1)	56 (50.9)	315 (39.4)	1.16×10^−48^	4.41		(3.59–5.41)	0.0229	1.60		(1.07–2.38)
D70	292 (31.8)	55 (50.0)	434 (54.3)	5.78×10^−21^	0.39		(0.32–0.48)	0.4160	0.84		(0.57–1.26)
I67	421 (45.8)	57 (51.8)	501 (62.6)	3.66×10^−12^	0.50		(0.42–0.61)	0.0364	0.64		(0.43–0.96)
S1	309 (33.6)	35 (31.8)	375 (46.9)	2.31×10^−8^	0.57		(0.47–0.70)	0.0030	0.53		(0.35–0.81)
DERAA	68 (7.4)	15 (13.6)	134 (16.8)	2.05×10^−9^	0.40		(0.29–0.54)	0.4922	0.78		(0.44–1.40)

ACPA: anti-citrullinated peptide antibody, ACPA(+): ACPA-positive, ACPA(−): ACPA-negative, RA: rheumatoid arthritis, OR: odds ratio, CI: confidence interval, *P*c: corrected *P* value, NS: not significant. Allele carrier frequencies are shown in parenthesis (%). Association was tested by Fisher's exact test using 2×2 contingency tables. Allele groups SE, D70, I67, S1, and DERAA were as defined in the Materials and Methods section.

The predisposing association was also confirmed between SE and ACPA(−) RA (*P* = 0.0229, OR 1.60, 95% CI 1.07–2.38), albeit weakly. A tendency towards a positive association of *04:05 and *14:54 with ACPA(−) RA was observed (*04:05: *P* = 0.0126, *P*c = 0.3667, OR 1.75, 95% CI 1.15–2.69;*14:54: *P* = 0.0202, OR 2.25, *P*c = 0.5861, 95% CI 1.17–4.32). On the other hand, DRB1*15:02 was negatively associated with ACPA(−) RA (*P* = 8.87×10^−5^, *P*c = 0.0026, OR 0.26, 95% CI 0.12–0.56).

We next examined associations of alleles other than SE with ACPA(+) and ACPA(−) RA stratified by the presence or absence of SE (Table 6). This analysis showed that DRB1*13:02 and D70 were negatively associated with ACPA(+) RA in both SE-positive and -negative subjects (SE-negative: *P* = 0.0212, OR 0.59, 95% CI 0.38–0.92; SE-positive: *P* = 0.0144, OR 0.53, 95% CI 0.32–0.87 and SE-negative: *P* = 0.0011, OR 0.59, 95% CI 0.43–0.81: SE-positive, *P* = 0.0001, OR 0.56, 95% CI 0.42–0.75, respectively). D70 alleles other than *13:02 were protectively associated with ACPA(+) RA in SE-positive (*P* = 0.0076, OR 0.65, 95% CI 0.47–0.89), but not in SE-negative subjects. These data suggest that the negative association of D70 alleles with ACPA(+) RA in SE-negative patients is mainly mediated by *13:02. Thus, the negative association of *13:02 with ACPA(+) RA was confirmed in SE-negative and -positive subjects.

**Table 6:** *HLA-DRB1* allele carrier frequency in ACPA(+) and ACPA(−) RA patients and controls stratified for the presence of SE.

		ACPA(+) RA	ACPA(−) RA	Control	ACPA(+) RA			ACPA(−) RA		
		(n = 919)	(n = 110)	(n = 800)	*P*	OR	95%CI	*P*	OR	95%CI
*03	SE negative	0 (0.0)	1 (1.9)	0 (0.0)	1.0000	2.04	(0.04–102.91)	0.1002	27.22	(1.10–676.68)
	SE positive	1 (0.1)	0 (0.0)	0 (0.0)	1.0000	1.39	(0.06–34.24)	1.0000	5.58	(0.11–284.33)
*04 other than SE	SE negative	24 (10.1)	5 (9.3)	81 (16.7)	0.0183	0.56	(0.34–0.91)	0.1755	0.51	(0.20–1.32)
	SE positive	33 (4.8)	5 (8.9)	34 (10.8)	0.0009	0.42	(0.26–0.69)	0.8156	0.81	(0.30–2.17)
*07	SE negative	4 (1.7)	0 (0.0)	5 (1.0)	0.4862	1.64	(0.44–6.17)	1.0000	0.80	(0.04–14.69)
	SE positive	3 (0.4)	0 (0.0)	2 (0.6)	0.6540	0.69	(0.12–4.17)	1.0000	1.11	(0.05–23.42)
*08	SE negative	57 (23.9)	17 (31.5)	140 (28.9)	0.1826	0.78	(0.54–1.11)	0.7523	1.13	(0.62–2.08)
	SE positive	50 (7.3)	4 (7.1)	41 (13.0)	0.0062	0.53	(0.34–0.82)	0.2704	0.51	(0.18–1.50)
*09	SE negative	129 (54.2)	20 (37.0)	161 (33.2)	8.63×10^−8^	2.38	(1.73–3.27)	0.6488	1.18	(0.66–2.12)
	SE positive	123 (18.1)	10 (17.9)	52 (16.5)	0.5916	1.11	(0.78–1.59)	0.8459	1.10	(0.52–2.32)
*11	SE negative	11 (4.6)	3 (5.6)	29 (6.0)	0.4942	0.76	(0.37–1.55)	1.0000	0.92	(0.27–3.14)
	SE positive	15 (2.2)	1 (1.8)	4 (1.3)	0.4558	1.75	(0.58–5.32)	0.5609	1.41	(0.16–12.89)
*12	SE negative	30 (12.6)	11 (20.4)	66 (13.6)	0.8157	0.92	(0.58–1.45)	0.2158	1.62	(0.80–3.31)
	SE positive	50 (7.3)	6 (10.7)	21 (6.7)	0.7915	1.11	(0.65–1.88)	0.2699	1.68	(0.65–4.37)
*13	SE negative	30 (12.6)	8 (14.8)	102 (21.0)	0.0056	0.54	(0.35–0.84)	0.3732	0.65	(0.30–1.43)
	SE positive	38 (5.6)	7 (12.5)	32 (10.2)	0.0110	0.52	(0.32–0.85)	0.6357	1.26	(0.53–3.02)
*14 other than SE	SE negative	28 (11.8)	15 (27.8)	89 (18.4)	0.0242	0.59	(0.38–0.94)	0.1031	1.71	(0.90–3.24)
	SE positive	51 (7.5)	6 (10.7)	33 (10.5)	0.1406	0.69	(0.44–1.10)	1.0000	1.03	(0.41–2.57)
*15	SE negative	100 (42.0)	13 (24.1)	207 (42.7)	0.8732	0.97	(0.71–1.33)	0.0085	0.43	(0.22–0.82)
	SE positive	148 (21.7)	9 (16.1)	55 (17.5)	0.1283	1.31	(0.93–1.85)	1.0000	0.91	(0.42–1.96)
*16	SE negative	6 (2.5)	0 (0.0)	9 (1.9)	0.5838	1.37	(0.48–3.89)	0.6090	0.46	(0.03–8.02)
	SE positive	4 (0.6)	2 (3.6)	6 (1.9)	0.0813	0.30	(0.09–1.09)	0.3460	1.91	(0.38–9.70)
*13:01	SE negative	0 (0.0)	0 (0.0)	7 (1.4)	0.1024	0.13	(0.01–2.35)	1.0000	0.59	(0.03–10.39)
	SE positive	1 (0.1)	1 (1.8)	1 (0.3)	0.5327	0.46	(0.03–7.41)	0.2794	5.71	(0.35–92.64)
*13:02	SE negative	30 (12.6)	8 (14.8)	95 (19.6)	0.0212	0.59	(0.38–0.92)	0.4690	0.71	(0.33–1.56)
	SE positive	37 (5.4)	6 (10.7)	31 (9.8)	0.0144	0.53	(0.32–0.87)	0.8102	1.10	(0.44–2.77)
*15:01	SE negative	41 (17.2)	9 (16.7)	89 (18.4)	0.7578	0.93	(0.62–1.39)	0.8542	0.89	(0.42–1.89)
	SE positive	65 (9.5)	6 (10.7)	18 (5.7)	0.0481	1.74	(1.01–2.99)	0.2315	1.98	(0.75–5.23)
*15:02	SE negative	62 (26.1)	4 (7.4)	131 (27.0)	0.8580	0.95	(0.67–1.35)	0.0008	0.22	(0.08–0.61)
	SE positive	83 (12.2)	3 (5.4)	37 (11.7)	0.9167	1.04	(0.69–1.58)	0.2395	0.43	(0.13–1.43)
D70	SE negative	127 (53.4)	35 (64.8)	320 (66.0)	0.0011	0.59	(0.43–0.81)	0.8804	0.95	(0.53–1.71)
	SE positive	165 (24.2)	20 (35.7)	114 (36.2)	0.0001	0.56	(0.42–0.75)	1.0000	0.98	(0.54–1.77)
I67	SE negative	168 (70.6)	32 (59.3)	371 (76.5)	0.1018	0.74	(0.52–1.05)	0.0080	0.45	(0.25–0.80)
	SE positive	253 (37.2)	25 (44.6)	130 (41.3)	0.2338	0.84	(0.64–1.11)	0.6610	1.15	(0.65–2.03)
S1	SE negative	123 (51.7)	19 (35.2)	288 (59.4)	0.0552	0.73	(0.54–1.00)	0.0008	0.37	(0.21–0.67)
	SE positive	186 (27.3)	16 (28.6)	87 (27.6)	0.9392	0.98	(0.73–1.33)	0.8724	1.05	(0.56–1.97)
D70 other than *13:02	SE negative	110 (46.2)	29 (53.7)	253 (52.2)	0.1542	0.79	(0.58–1.08)	0.8863	1.06	(0.61–1.87)
	SE positive	128 (18.8)	14 (25.0)	83 (26.3)	0.0076	0.65	(0.47–0.89)	1.0000	0.93	(0.48–1.79)
I67 other than *13:02	SE negative	156 (65.5)	27 (50.0)	310 (63.9)	0.6802	1.07	(0.78–1.49)	0.0538	0.56	(0.32–0.99)
	SE positive	216 (31.7)	19 (33.9)	99 (31.4)	0.9417	1.01	(0.76–1.35)	0.7560	1.12	(0.61–2.05)
S1 other than *13:02	SE negative	100 (42.0)	13 (24.1)	213 (43.9)	0.6329	0.93	(0.68–1.27)	0.0055	0.40	(0.21–0.77)
	SE positive	149 (21.9)	10 (17.9)	56 (17.8)	0.1520	1.30	(0.92–1.82)	1.0000	1.01	(0.48–2.11)
I67 other than *15:02	SE negative	123 (51.7)	30 (55.6)	290 (59.8)	0.0455	0.72	(0.53–0.98)	0.5617	0.84	(0.48–1.48)
	SE positive	170 (25.0)	22 (39.3)	93 (29.5)	0.1420	0.79	(0.59–1.07)	0.1594	1.54	(0.86–2.78)
S1 other than *15:02	SE negative	67 (28.2)	17 (31.5)	180 (37.1)	0.0194	0.66	(0.47–0.93)	0.4590	0.78	(0.43–1.42)
	SE positive	103 (15.1)	13 (23.2)	50 (15.9)	0.7771	0.94	(0.65–1.36)	0.1799	1.60	(0.80–3.19)

ACPA: anti-citrullinated peptide antibody, ACPA(+): ACPA-positive, ACPA(−): ACPA-negative, RA: rheumatoid arthritis, SE: Shared epitope, OR: odds ratio, CI: confidence interval, Allele carrier frequencies are shown in parenthesis (%). Association was tested by Fisher's exact test using 2×2 contingency tables. SE negative: “*A*/*A*” or “*A*/other than SE or *A*” vs. “other than SE or *A*/other than SE or *A*”. SE positive: “SE/*A*” vs. “SE/other than *A*”. Allele groups SE, I67, D70, and S1 were as defined in the Materials and Methods section.

The DRB1*15:02 allele was negatively associated with ACPA(−) RA in SE-negative people (*P* = 0.0008, OR 0.22, 95% CI 0.08–0.61). I67 and S1 alleles were negatively associated with ACPA(−) RA in SE-negative subjects (*P* = 0.0080, OR 0.45, 95% CI 0.25–0.80 and *P* = 0.0008, OR 0.37, 95% CI 0.21–0.67, respectively). However, I67 alleles other than *15:02 or S1 alleles other than *15:02 were not associated with ACPA(−) RA. These data suggest that the negative associations of I67 and S1 with ACPA(−) RA in SE-negative subjects are mainly mediated by *15:02. Thus, the negative association of *15:02 with ACPA(−) RA was detected in SE-negative people.

Then we examined the protective effects of *13:02 against ACPA(+) RA in the presence of predisposing alleles for ACPA(+) RA, DRB1*04:05 and *09:01 (Table 5). As shown in Table 7, the risk for RA was decreased when these alleles were present together with *13:02 (*04:05: *P* = 0.0202, OR = 0.49, 95% CI 0.27–0.88; *09:01: *P* = 0.0035, OR = 0.30, 95% CI 0.13–0.69).

**Table 7:** *HLA-DRB1* genotype frequency in ACPA(+) and ACPA(−) RA patients and controls.

	ACPA(+) RA (n = 919)	Control (n = 800)	*P*	OR	95%CI
*04:05/*13:02	28 (5.9)	21 (11.4)	0.0202	0.49	(0.27–0.88)
*09:01/*13:02	8 (3.2)	21 (9.9)	0.0035	0.30	(0.13–0.69)
*04:01/*13:02	3 (4.6)	2 (11.8)	0.2755	0.36	(0.06–2.37)
*01:01/*13:02	4 (2.7)	3 (3.6)	0.7061	0.75	(0.16–3.44)
*14:54/*13:02	0 (0.0)	6 (13.3)	0.0263	0.07	(0.00–1.28)
	ACPA(−) RA (n = 110)	Control (n = 800)	*P*	OR	95%CI
*04:05/*15:02	2 (5.3)	23 (12.4)	0.2665	0.39	(0.09–1.74)
*09:01/*15:02	1 (3.3)	28 (13.1)	0.2228	0.23	(0.03–1.74)
*04:01/*15:02	1 (25.0)	1 (5.9)	0.3524	5.33	(0.26–110.80)
*01:01/*15:02	0 (0.0)	5 (6.0)	1.0000	0.68	(0.04–13.19)
*14:54/*15:02	1 (7.7)	7 (15.6)	0.6686	0.45	(0.05–4.06)

ACPA: anti-citrullinated peptide antibody, ACPA(+): ACPA-positive, ACPA(−): ACPA-negative, RA: rheumatoid arthritis, SE: Shared epitope, OR: odds ratio, CI: confidence interval, Allele carrier frequencies are shown in parenthesis (%). Association was tested by Fisher's exact test using 2×2 contingency tables. Upper row: “*B*/*13:02” vs. “*B*/other than *13:02”. Lower row: “*B*/*15:02” vs. “*B*/other than *15:02”.

The protective effects of the *15:02 allele against ACPA(−) RA were also analyzed in the presence of predisposing alleles (Table 7). DRB1*04:05 and *14:54 are potentially risk alleles for ACPA(−) RA (Table 5). The risk for ACPA(−) RA showed tendency towards decrease when these alleles were present together with *15:02 (*04:05: *P* = 0.2665, OR = 0.39; *14:54: *P* = 0.6686, OR = 0.45), but these differences were not statistically significant.

### Certain amino acid residues in the HLA-DRβ chain are associated with RA

**Figure 1:**
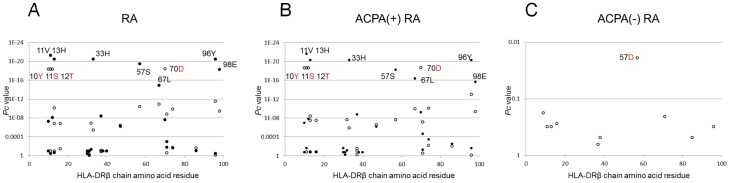
Associations of amino acid residues in the DRβ chain with RA (A), ACPA-positive [ACPA(+)] RA (B), and ACPA-negative [ACPA(−)] RA (C). Corrected *P* (*P*c) values were calculated by multiplying the *P* value by the number of amino acid residues tested. Associations were established by Fisher's exact test using 2×2 contingency tables. Positive associations are indicated by filled circles and negative associations by open circles.

Finally, we analyzed the association with RA with respect to each amino acid residue in the HLA-DRβ chain. Tyrosine at position 10 (10Y, *P* = 1.34×10^−20^, OR = 0.44, *P*c = 4.59×10^−19^, 95% CI 0.37–0.52), serine at position 11 (11S, *P* = 1.35×10^−20^, OR = 0.44, *P*c = 4.59×10^−19^, 95% CI 0.37–0.52), threonine at position 12 (12T, *P* = 1.35×10^−20^, OR = 0.44, *P*c = 4.59×10^−19^, 95% CI 0.37–0.52), and aspartic acid at position 70 (70D, *P* = 1.15×10^−20^, OR = 0.43, *P*c = 3.91×10^−19^, 95% CI 0.36–0.52) in the DRβ chain showed strong protective associations with RA (Figure 1A, open circles). Similar associations were observed with ACPA(+) RA (Figure 1B), whereas aspartic acid at position 57 (57D, *P* = 0.0006, OR = 0.46, *P*c = 0.0191, 95% CI 0.30–0.71) in the DRβ chain showed a slight protective association with ACPA(−) RA (Figure 1C). Thus, association analysis suggested roles for specific amino acid residues in the HLA-DRβ chain.

## Discussion

Many groups have investigated associations between *HLA-DRB1* alleles and RA disease susceptibility. However, few studies have focused on protective effects of *DRB1* alleles against RA [11], [12]. In the present study, we determined that the DRB1*13:02 allele plays a protective role in Japanese RA, especially in ACPA(+) RA, using RPE analysis (Table 2). A lower frequency of *13:02 alleles in Asian patients with RA has been reported before [13], [14], [15], [16], [17]. In the genotype analysis, lower frequencies of the “HLA-DRB1*04:05/*13:02”, or “*09:01/*13:02” genotypes in RA were observed (Table 4). Thus, the protective effects of *13:02 seem to overcome the predisposing effects of *04:05 or *09:01. Several studies have shown that certain *DRB1* alleles are negatively associated with RA and also some negatively associated allele groups defined by amino acid sequences, such as D70, I67, S1 and DERAA (Table 3) [6], [7], [8], [9], [10]. Our results indicated that the protective effects of these allele groups were mainly attributable to *13:02 in Japanese RA, whereas they are attributable to *13:01 in European RA [12]. The age at onset of *13:02 allele carriers was higher than non-carriers (Table S3), suggesting that the allele carriers of *13:02 may be associated with RA subsets with higher age at onset, and/or the age at onset may be delayed in the presence of the *13:02 allele.

DRB1*13:02 commonly belongs to the haplotype DRB1*13:02-DQB1*06:04-DPB1*04:01, which shows evidence for positive selection in Japanese in recent history [27]. The DRB1*13:02 allele is also a protective allele for cervical cancer [28], autoimmune hepatitis [29], and DPB1*04:01 is protective for hepatitis B infection [30]. Certain genes of this haplotype could be protective for these diseases, in addition to RA.

It was reported that SE alleles are strongly associated with ACPA(+) RA, but weakly with ACPA(−) RA [1], and this was confirmed in the present study. We documented protective effects of DRB1*13:02 against ACPA(+) RA and DRB1*15:02 against ACPA(−) RA in Japanese. Although the sample size of ACPA(−) RA is not large enough, the protective effect of *15:02 against ACPA(−) RA was also reported in another study [31], supporting the results. These findings could be explained by differences in the pathogenesis of ACPA(+) and ACPA(−) RA. Although the genotype of DRB1*03/*13 was reported to be associated with ACPA(−) RA in a European population [11], such an association was not found in the current study.

Amino acid residues 10Y, 11S, 12T, and 70D of the HLA-DRβ chain were negatively associated with RA (Figure 1A). Amino acid residues 11 and 70 form the HLA-DR peptide-binding groove [32]. These data suggest the involvement of peptide antigens bound to specific HLA molecules in controlling the development of RA. Associations of amino acid residues 10, 11, 12, 13, 33, 37, 47, 67, 70, 96 and 98 of the HLA-DRβ chain were reported in European ACPA(+) RA [33], showing slightly different association pattern from the results of this study (Figure 1B). However, associated amino acid residues 10, 11, 12, 13, 33, 57, 70, 96 and 98 of HLA-DRβ chain in Korean ACPA(+) RA [33] were more similar to the results (Figure 1B), reflecting the difference of DRB1 allele frequencies between European and Asian populations.

The negative association with the DRB1*13:02 allele needs to be confirmed in future independent studies. Because the distribution of *HLA* alleles in other ethnic populations is different from the Japanese, the protective role of some *DRB1* alleles in RA in other populations should be determined.

Thus, the present study identified a negative association of DRB1*13:02 with Japanese RA; our findings support the protective role of DRB1*13:02 alleles in the pathogenesis of ACPA(+) RA.

## Supporting Information

Table S1 ***HLA-DRB1*** **homozygous frequency in the RA patients and controls.**
(PDF)

Table S2 ***HLA-DRB1*** **allele frequency in the RA patients and controls.**
(PDF)

Table S3 **Age at onset of** ***HLA-DRB1*** **allele carrier or non-carrier in the RA patients.**
(PDF)
