# Supplementary material for: Protective Effect of the HLA-DRB1*13:02 Allele in Japanese Rheumatoid Arthritis Patients
Source: PLoS One. 2014 Jun 9;9(6):e99453. doi: 10.1371/journal.pone.0099453 (PMC4049831; doi:10.1371/journal.pone.0099453)
Supplement: Table S1 — HLA-DRB1 homozygous frequency in the RA patients and controls. (PDF) [file pone.0099453.s001.pdf]

Supplementary Table S1. *HLA-DRB1* homozygous frequency in the RA patients and controls.

	Case (n=1480)	Control (n=800)	*P*	OR	*P*c	95%CI
*DRB1*01:01*	9 (0.6)	1 (0.1)	0.1801	4.89	NS	(0.62-38.66)
*DRB1*04:01*	2 (0.1)	1 (0.1)	1.0000	1.08	NS	(0.10-11.94)
*DRB1*04:03*	2 (0.1)	0 (0.0)	0.5443	2.71	NS	(0.13-56.46)
*DRB1*04:05*	79 (5.3)	6 (0.8)	$1.04X10^{-9}$	7.46	$1.87X10^{-8}$	(3.24-17.19)
*DRB1*04:06*	1 (0.1)	0 (0.0)	1.0000	1.62	NS	(0.07-39.89)
*DRB1*04:10*	2 (0.1)	0 (0.0)	0.5443	2.71	NS	(0.13-56.46)
*DRB1*08:03*	9 (0.6)	3 (0.4)	0.5579	1.63	NS	(0.44-6.02)
*DRB1*09:01*	51 (3.4)	8 (1.0)	0.0003	3.53	0.0047	(1.67-7.48)
*DRB1*10:01*	1 (0.1)	0 (0.0)	1.0000	1.62	NS	(0.07-39.89)
*DRB1*11:01*	1 (0.1)	0 (0.0)	1.0000	1.62	NS	(0.07-39.89)
*DRB1*12:01*	2 (0.1)	1 (0.1)	1.0000	1.08	NS	(0.10-11.94)
*DRB1*12:02*	1 (0.1)	0 (0.0)	1.0000	1.62	NS	(0.07-39.89)
*DRB1*13:02*	5 (0.3)	5 (0.6)	0.3346	0.54	NS	(0.16-1.87)
*DRB1*14:03*	1 (0.1)	0 (0.0)	1.0000	1.62	NS	(0.07-39.89)
*DRB1*14:06*	1 (0.1)	0 (0.0)	1.0000	1.62	NS	(0.07-39.89)
*DRB1*14:54*	1 (0.1)	0 (0.0)	1.0000	1.62	NS	(0.07-39.89)
*DRB1*15:01*	10 (0.7)	3 (0.4)	0.5614	1.81	NS	(0.50-6.59)
*DRB1*15:02*	11 (0.7)	3 (0.4)	0.4025	1.99	NS	(0.55-7.15)

RA: rheumatoid arthritis, OR: odds ratio, CI: confidence interval, *P*c: corrected *P* value, NS: not significant,
Homozygous frequencies are shown in parenthesis (%).
Association was tested by Fisher's exact test using 2X2 contingency tables.
