# Supplementary material for: Protective Effect of the HLA-DRB1*13:02 Allele in Japanese Rheumatoid Arthritis Patients
Source: PLoS One. 2014 Jun 9;9(6):e99453. doi: 10.1371/journal.pone.0099453 (PMC4049831; doi:10.1371/journal.pone.0099453)
Supplement: Table S2 — HLA-DRB1 allele frequency in the RA patients and controls. (PDF) [file pone.0099453.s002.pdf]

Supplementary Table S2. *HLA-DRB1* allele frequency in the RA patients and controls.

	Case (2n=2960)	Control (2n=1600)	*P*	OR	*P*c	95%CI
*DRB1*01:01*	219 (7.4)	86 (5.4)	0.0090	1.41	0.2803	(1.09-1.82)
*DRB1*03:01*	2 (0.1)	0 (0.0)	0.5444	2.70	NS	(0.13-56.38)
*DRB1*04:01*	86 (2.9)	18 (1.1)	$6.67X10^{-5}$	2.63	0.0021	(1.58-4.39)
*DRB1*04:03*	40 (1.4)	45 (2.8)	0.0008	0.47	0.0242	(0.31-0.73)
*DRB1*04:04*	5 (0.2)	4 (0.3)	0.7283	0.68	NS	(0.18-2.52)
*DRB1*04:05*	817 (27.6)	197 (12.3)	$1.95X10^{-34}$	2.72	$6.04X10^{-33}$	(2.29-3.22)
*DRB1*04:06*	59 (2.0)	60 (3.8)	0.0006	0.52	0.0186	(0.36-0.75)
*DRB1*04:07*	4 (0.1)	15 (0.9)	0.0001	0.14	0.0036	(0.05-0.43)
*DRB1*04:10*	72 (2.4)	21 (1.3)	0.0112	1.87	0.3478	(1.15-3.06)
*DRB1*07:01*	10 (0.3)	7 (0.4)	0.6161	0.77	NS	(0.29-2.03)
*DRB1*08:02*	56 (1.9)	62 (3.9)	0.0001	0.48	0.0036	(0.33-0.69)
*DRB1*08:03*	144 (4.9)	132 (8.3)	$8.63X10^{-6}$	0.57	0.0003	(0.45-0.73)
*DRB1*08:09*	1 (0.0)	2 (0.1)	0.2829	0.27	NS	(0.02-2.98)
*DRB1*08:23*	1 (0.0)	0 (0.0)	1.0000	1.62	NS	(0.07-39.85)
*DRB1*09:01*	474 (16.0)	233 (14.6)	0.1988	1.12	NS	(0.94-1.33)
*DRB1*10:01*	26 (0.9)	2 (0.1)	0.0011	7.08	0.0337	(1.68-29.87)
*DRB1*11:01*	41 (1.4)	33 (2.1)	0.0868	0.67	NS	(0.42-1.06)
*DRB1*12:01*	97 (3.3)	60 (3.8)	0.3964	0.87	NS	(0.63-1.21)
*DRB1*12:02*	51 (1.7)	29 (1.8)	0.8142	0.95	NS	(0.60-1.50)
*DRB1*13:01*	5 (0.2)	8 (0.5)	0.0756	0.34	NS	(0.11-1.03)
*DRB1*13:02*	112 (3.8)	134 (8.4)	$2.39X10^{-10}$	0.43	$7.40X10^{-9}$	(0.33-0.56)
*DRB1*14:02*	2 (0.1)	0 (0.0)	0.5444	2.70	NS	(0.13-56.38)
*DRB1*14:03*	33 (1.1)	38 (2.4)	0.0016	0.46	0.0483	(0.29-0.74)
*DRB1*14:04*	0 (0.0)	3 (0.2)	0.0431	0.08	NS	(0.00-1.49)
*DRB1*14:05*	28 (0.9)	35 (2.2)	0.0012	0.43	0.0377	(0.26-0.70)
*DRB1*14:06*	46 (1.6)	22 (1.4)	0.7018	1.13	NS	(0.68-1.89)
*DRB1*14:07*	2 (0.1)	2 (0.1)	0.6160	0.54	NS	(0.08-3.84)
*DRB1*14:54*	77 (2.6)	45 (2.8)	0.7008	0.92	NS	(0.64-1.34)
*DRB1*15:01*	188 (6.4)	116 (7.3)	0.2629	0.87	NS	(0.68-1.10)
*DRB1*15:02*	244 (8.2)	175 (10.9)	0.0031	0.73	0.0962	(0.60-0.90)
*DRB1*16:02*	17 (0.6)	15 (0.9)	0.1927	0.61	NS	(0.30-1.23)

RA: rheumatoid arthritis, OR: odds ratio, CI: confidence interval, *P*c: corrected *P* value, NS: not significant,
Allele frequencies are shown in parenthesis (%).
Association was tested by Fisher's exact test using 2X2 contingency tables.
