# Supplementary material for: Protective Effect of the HLA-DRB1*13:02 Allele in Japanese Rheumatoid Arthritis Patients
Source: PLoS One. 2014 Jun 9;9(6):e99453. doi: 10.1371/journal.pone.0099453 (PMC4049831; doi:10.1371/journal.pone.0099453)
Supplement: Table S3 — Age at onset of HLA-DRB1 allele carrier or non-carrier in the RA patients. (PDF) [file pone.0099453.s003.pdf]

Supplementary Table S3. Age at onset of *HLA-DRB1* allele carrier or non-carrier in the RA patients.

	carrier		non-carrier		
	n	age at onset	n	age at onset	*P*
SE	719	49.2 (14.0)	280	49.7 (15.3)	0.5208
**04:05*	510	48.2 (13.8)	489	50.5 (14.9)	0.0070
**13:02*	83	53.8 (14.0)	916	48.9 (14.4)	0.0027
**04:01*	67	48.6 (13.2)	932	49.4 (14.5)	0.6372
**09:01*	281	48.6 (14.7)	718	49.6 (14.2)	0.3725
**01:01*	143	52.9 (13.3)	856	48.7 (14.5)	0.0021

RA: rheumatoid arthritis. Average values of each group are shown.
Standard deviations are shown in parenthesis. Differences were tested by Mann-Whitney's U test.
